# Body composition and cognitive function in Chinese rural adults: an exploratory factor analysis and network analysis

**DOI:** 10.3389/fnagi.2025.1722050

**Published:** 2025-12-18

**Authors:** Lei Wang, Hongjuan Liu, Xianfeng Meng, Zhengjiao Tuo, Yuning Zhou, Peiyi Wu, Enhui Wang, Yuxin Shen, Ziyi Wang, Caijiu Deng, Yuang Liu, Yanqing Tang, Yifang Zhou

**Affiliations:** 1 School of Public Health, China Medical University, Shenyang, China; 2 Department of Psychiatry, Shengjing Hospital of China Medical University, Shenyang, China; 3 The Second Clinical College, China Medical University, Shenyang, China; 4 Liaoning Provincial Mental Health Center, Kaiyuan, China

**Keywords:** body composition, cognitive function, exploratory factor analysis, network analysis, rural adults

## Abstract

**Background:**

Growing evidence suggests that body composition has a significant influence on cognitive function. However, their relationship remains controversial. This study investigated the association between body composition and cognitive function.

**Methods:**

This multicenter cross-sectional study recruited participants from 38 rural townships in Beizhen from July to August 2023. We included participants who completed both cognitive function assessments and body composition measurements. Exploratory factor analysis was employed for dimensionality reduction and classification of body composition. A logistic regression model was utilized to evaluate the association between primary body composition and cognitive decline. Network analysis was performed using R software to construct network models of body composition and cognitive function, to identify key variables and their interconnections.

**Results:**

Exploratory factor analysis classified 27 body composition variables into 6 factors. Among the 6 factors, “muscle mass” (OR = 0.393), “central obesity” (OR = 1.69), and “leg-dominant fat distribution” (OR = 0.473) are associated with cognitive function. “Muscle mass,” “central obesity,” and “leg-dominant fat distribution” were used to construct network models related to cognitive function. In these three models, the most central domains are all language, attention, and registration.

**Conclusion:**

This study found that “central obesity” increased the risk of cognitive decline, while “muscle mass” and “leg-dominant fat distribution” had protective effects. Interventions targeting language, attention, and registration domains might help address cognitive decline caused by changes in body composition.

## Introduction

Dementia is a syndrome characterized by acquired and persistent cognitive decline that can impair cognitive domains such as memory, thinking, orientation, comprehension, calculation, learning, language, and judgment. These symptoms have a severe impact on patients’ quality of life (Gale et al., 2018). In 2019, there were approximately 55.2 million dementia patients globally, with over 60% living in low- to middle-income countries (World Health Organization, 2021). As the largest middle-income country, China reported a dementia prevalence of 6.0% by the end of 2018, amounting to about 15.07 million individuals (Jia et al., 2020a). According to the existing survey results, China has become the country with the highest number of dementia patients globally, which not only seriously undermines the quality of life for patients but also imposes significant economic and social burdens on the public health system (Jia et al., 2020b). However, effective treatment options for dementia are still lacking (Sikkes et al., 2021). Developing preventive strategies that target modifiable risk factors for the onset of disease is essential for delaying or preventing the onset and progression of cognitive impairment.

For rural areas, early intervention in cognitive impairment is of great significance (Wang et al., 2020). Recent studies have confirmed a higher risk of cognitive impairment among rural residents compared to their urban residents (Lu et al., 2021; Qin et al., 2022). Rural areas are constrained by healthcare and educational resources, with cognitive impairments usually identified only at the advanced stages of the disease, a factor that imposes a substantial disease burden. Furthermore, the lower levels of health literacy in the rural population put them at a disadvantage in adopting healthy lifestyles and implementing preventive measures. Consequently, research into modifiable risk factors for cognitive impairment may hold even greater importance in rural settings than in urban ones.

Body composition encompasses the various components of the body, including fat, muscle, bone, water, and minerals. Traditionally, most researchers have used Body Mass Index (BMI) as a surrogate measure of body composition when investigating its association with cognitive function (Ren et al., 2021; West et al., 2021; Liang et al., 2022; Dong et al., 2023; Shang et al., 2023; Li J. et al., 2024; Gwizdala et al., 2025). However, controversy remains in the research regarding the relationship between BMI and cognitive impairment (Liang et al., 2022; Dong et al., 2023; Shang et al., 2023). One reason for these discrepancies may be that BMI cannot distinguish between fat and muscle, which can mistakenly classify individuals with high muscle mass as having adipose tissue. However, different body components may play distinct roles in cognitive function. Empirical research has demonstrated a significant correlation between abdominal adiposity and the incidence of cognitive impairment (Liang et al., 2022; Liu et al., 2022; Li Q. et al., 2024; Uchida et al., 2024). Conversely, higher muscle mass has been associated with a reduced risk of cognitive decline (Noh et al., 2017; Lu et al., 2024; Wang et al., 2024; Zhan et al., 2025).

Besides, using a single indicator to measure changes in body composition often leads to inconsistent results (Beeri et al., 2021; Merchant et al., 2021; Chen et al., 2024; Wang et al., 2024), because a single indicator cannot fully capture the complex interactions among different body compositions. For instance, previous research has also indicated that the interplay between low muscle mass and high adipose tissue is associated with cognitive decline and dementia (Someya et al., 2022). Therefore, exploring interactions among body composition is necessary to study the relationship between body composition and cognitive function.

Exploratory factor analysis (EFA) can be used to identify the potential interactions among various indicators of body composition. Its primary purpose is to uncover the underlying factors in the data, simplify the data, and facilitate an understanding of the interactions among variables (Ringnér, 2008). Recent research has applied this approach to characterize high-dimensional datasets (e.g., gut microbiota profiles and body composition patterns) (Leong et al., 2020; Xu et al., 2024). Beyond enabling dimensionality reduction and classification, it facilitates the interpretation of interactive relationships among indicators.

Cognitive function encompasses multiple cognitive domains, including orientation, registration, attention, recall, and language abilities (Truong et al., 2024). Currently, there is a lack of research exploring the relationships between cognitive domains and body composition, as well as the key variables that underlie these relationships. Network analysis is a powerful method that has two main advantages. First, it can thoroughly analyze data to reveal the extent of interrelationships among various variables within the network, facilitating visualization (Epskamp et al., 2012). Second, it can identify key variables within complex networks, determining which critical domains could be targeted for intervention to alleviate cognitive impairment (Wang et al., 2023).

This study collected body composition data and cognitive function data in Beizhen City, Liaoning Province. We employed exploratory factor analysis to reduce and classify the body composition data. Additionally, we will use logistic regression to examine the relationship between body composition and cognitive function. Furthermore, network analysis will be conducted to explore the key variables in this relationship. This research will provide a theoretical foundation for future early screening of cognitive impairment in high-risk populations and offer personalized intervention guidance.

## Methods

### Participants

This study employed a cross-sectional design and used a cluster sampling method to select research subjects from 38 rural towns in Beizhen City between July and August 2023. Selection criteria included the following items: (1) Age ≥18 years; (2) Residing in the survey site for more than ≥6 months; (3) Respondents are informed and willing to participate in the study, and can cooperate with the investigator to complete the scale assessment and body composition measurement; (4) No severe visual or hearing impairment; (5) No acute or terminal stages of diseases such as severe heart disease, liver disease, kidney disease, lung disease, blood disease; (6) No mental disorders that may affect cognitive function.

### Sample size

A cross-sectional study using the Mini-Mental State Examination (MMSE) to estimate cognitive impairment revealed a prevalence rate of 26.07% among rural elderly in China (Qin et al., 2022). According to the cross-sectional sample size formula:


$$N=\frac{Z_{\alpha /2^{2}}p(1-p)}{d^{2}}$$

In this study, *p* = 0.2607. To ensure precision, *d* = 0.2*p* and α = 0.05 were selected. Calculations yielded a required sample size of approximately 275 participants. Given the multi-stage cluster sampling method employed, which carries higher sampling error, and considering potential low response rates, the sample size was increased by 50% compared to simple random sampling. The final minimum sample size was thus determined to be 504 participants. The study ultimately enrolled 793 participants, meeting the sample size requirement.

### Covariates

A questionnaire survey was conducted through face-to-face interviews. The questionnaire included basic information (age, gender, education), lifestyle history (smoking, alcohol use), sleep status, depressive symptoms, and a history of previous chronic diseases (hypertension, diabetes, cardiovascular disease, thyroid disease, rheumatic arthritis).

### Body composition measurement

The measurements are performed using well-calibrated instruments. Height, waist circumference, and hip circumference are all measured manually. The grip strength was measured using the CAMRY EH101 electronic grip dynamometer. Whole-body and localized (arms, trunk, and legs) fat mass and muscle mass, and body weight were assessed by bioimpedance using the Omron HBF702T body composition analyzer. A total of 27 body composition items and their derived results were measured. The specific items are shown in Supplementary Table S1.

### Cognitive assessment

MMSE evaluates cognitive function and is ideal for conducting extensive epidemiological surveys due to its brevity, rapidity, and good reliability and validity (Creavin et al., 2016). In this study, we used Zhang Yuan’s revised Chinese version of the MMSE to assess cognitive function (Wu et al., 2022). The MMSE primarily assesses five dimensions: orientation, registration, attention, recall, and language, comprising a total of 30 items. Each item is scored on a scale of 0-1points, with illiterate individuals scoring ≤17 points, primary school students scoring ≤20 points, and middle school and above scoring ≤24 points, assessed as cognitive decline (Li et al., 2016; Dong et al., 2023; Shang et al., 2023).

### Statistical analysis

SPSS 27.0 software was used to analyze the baseline data statistically. The univariate analysis was conducted by chi-square test, and the meaningful variables (*p* ≤ 0.1) of the univariate analysis were included as covariates in the regression analysis.

SPSS was used to perform exploratory factor analysis of 27 body components. Principal Component Analysis (PCA) (Ringnér, 2008) was combined with the maximum variance rotation method (Nordmann et al., 2005) for EFA. Under the assumption that the characteristic value ≥1 and the load ≥0.4 apply to each factor, the number and composition of the factors were determined using lithotripsy diagrams. Each factor is named after its predominant body composition characteristics. According to the quartile method, the obtained factors were divided into low (first quartile), medium (second quartile), and high (third quartile) exposure levels. The low exposure level was used as a reference, and the relationship between moderate or high exposure levels and cognitive decline was studied by logistic regression using SPSS, and OR and 95% CI were reported. Prior to constructing the logistic regression models, variance inflation factor (VIF) and Tolerance were used as core evaluation indicators to test for multicollinearity among all independent variables included in the models.

The cognitive function network model was constructed using the EBICglasso method within the bootnet package (v1.6) in RStudio 4.4.2 (Epskamp et al., 2018). The network model consists of “nodes” and “edges” that represent the study variables and their connections. In the network analysis, the combination of the Graph Minimum Absolute Shrinkage and Selection Operator (LASSO) and the Extended Bayesian Information Criterion (EBIC) was used to effectively reduce false positive connections and generate a more concise and interpretable network structure (Epskamp and Fried, 2018). Centrality is a key indicator of node importance, including strength centrality, closeness centrality, and betweenness centrality. Research indicates that betweenness and closeness are not suitable measures for node importance in psychological networks (Bringmann et al., 2019). Therefore, this study uses strength centrality to measure node importance, presenting the results as standardized Z-scores. A higher Z-score indicates that a node plays a more central role in the network and has a greater impact on cognitive function. The qgraph package (v1.9.8) was employed for visualizing networks. In addition, the study utilized the bootnet package (v1.6) to assess the stability of the network, with the Correlation Stability coefficient (CS) expected to be above 0.25 (Epskamp et al., 2018). The key steps of network analysis are provided in the Supplementary material.

In this study, the mice package (v3.17.0) in RStudio is used to impute missing data. Subgroup analysis is employed to investigate the impact of age and gender on the results, and sensitivity analysis is conducted to verify the rationality of body composition factor grouping and network analysis results.

## Results

### Sample characteristics

This study included 793 participants for analysis. All participants were recruited from rural areas. Among them, 215 (27.1%) were male and 578 (72.8%) were female. The age distribution was as follows: 217 (27.3%) participants were aged 55–59 years, and 576 (72.6%) were aged 60 years or older. In terms of educational attainment, 16 (2.0%) participants were illiterate, 342 (43.1%) had completed primary school, and 435 (54.9%) had received education at or above the middle school level. Regarding lifestyle factors, 198 (24.9%) participants were current smokers, and 144 (18.1%) were habitual drinkers. Additionally, 78 (9.8%) participants reported frequent insomnia, and 96 (12.1%) exhibited depressive symptoms. Regarding chronic diseases, 126 (15.8%) participants had hypertension, 47 (5.9%) had diabetes, 69 (8.7%) had cardiovascular disease, 26 (3.2%) had thyroid disease, and 46 (5.8%) had rheumatic arthritis. The study found that the prevalence of cognitive decline was 20.8%, with gender, age, education, cardiovascular disease, rheumatoid arthritis, and depressive symptoms participants more likely to experience cognitive decline (*p* ≤ 0.1). The characteristics of participants by cognitive function are shown in Table 1.

**Table 1:** Sample characteristics of participants (*N* = 793).

Characteristic	Cognitive decline		*χ*^2^	*p*-value
	No *n* (%)	Yes *n* (%)		
Age (years)				
<60	186 (85.7)	31 (14.3)	7.711	0.005
≥60	442 (76.7)	134 (23.3)		
Gender				
Male	161 (74.9)	54 (25.1)	3.324	0.068
Female	467 (80.8)	111 (19.2)		
Education				
Illiterate	10 (62.5)	6 (37.5)	19.868	<0.001
Primary School	295 (86.3)	47 (13.7)		
≥Middle School	323 (74.3)	112 (25.7)		
Smoking				
Yes	151 (76.3)	47 (23.7)	1.375	0.241
No	477 (80.2)	118 (19.8)		
Alcohol use				
Yes	111 (77.1)	33 (22.9)	0.475	0.491
No	517 (79.7)	132 (20.3)		
Hypertension				
Yes	100 (79.4)	26 (20.6)	0.003	0.959
No	528 (79.2)	139 (20.8)		
Diabetes				
Yes	36 (76.6)	11 (23.4)	0.205	0.651
No	592 (79.4)	154 (20.6)		
Cardiovascular disease				
Yes	60 (87)	9 (13)	2.764	0.096
No	568 (78.5)	156 (21.5)		
Thyroid disease				
Yes	21 (80.8)	5 (19.2)	0.041	0.840
No	607 (79.1)	160 (20.9)		
Rheumatic arthritis				
Yes	42 (91.3)	4 (8.7)	4.374	0.037
No	586 (78.4)	161 (21.6)		
Depressive symptoms				
Yes	70 (72.9)	26 (27.1)	2.564	0.100
No	556 (80)	139 (20)		
Insomnia				
Yes	59 (75.6)	19 (24.3)	0.662	0.416
No	569 (79.6)	146 (20.4)		

### Body composition

In this study, the KMO value = 0.802, and the Bartlett’s test of sphericity *p*-value = 0.000. Finally, twenty-seven body compositions were identified, six factors, and the cumulative contribution rate of variance of these factors was 88.836%. Factor 1 is “fat mass” (total body fat mass, arm fat mass, leg fat mass, trunk fat mass, etc.); Factor 2 is “muscle mass” (total body muscle mass, arm muscle mass, leg muscle mass, trunk muscle mass, etc.); Factor 3 is “central obesity” (cone index, body shape index, weight-adjusted waist circumference index, waist-to-hip ratio); Factor 4 is “muscle strength” (grip strength, grip strength/BMI, grip strength/arm muscle mass); Factor 5 is “leg-dominant muscle distribution” (emphasizing that the muscle mass are mainly distributed in the legs, and the fat mass is concentrated primarily on the trunk); Factor 6 is “leg-dominant fat distribution” (emphasizing that the fat mass is mainly concentrated in the legs, and the muscle mass are primarily concentrated in the limbs). The specific body composition indexes and the factor loading matrix after rotation are shown in Supplementary Table S2.

### Association of body composition with cognitive decline

**Figure 1:**
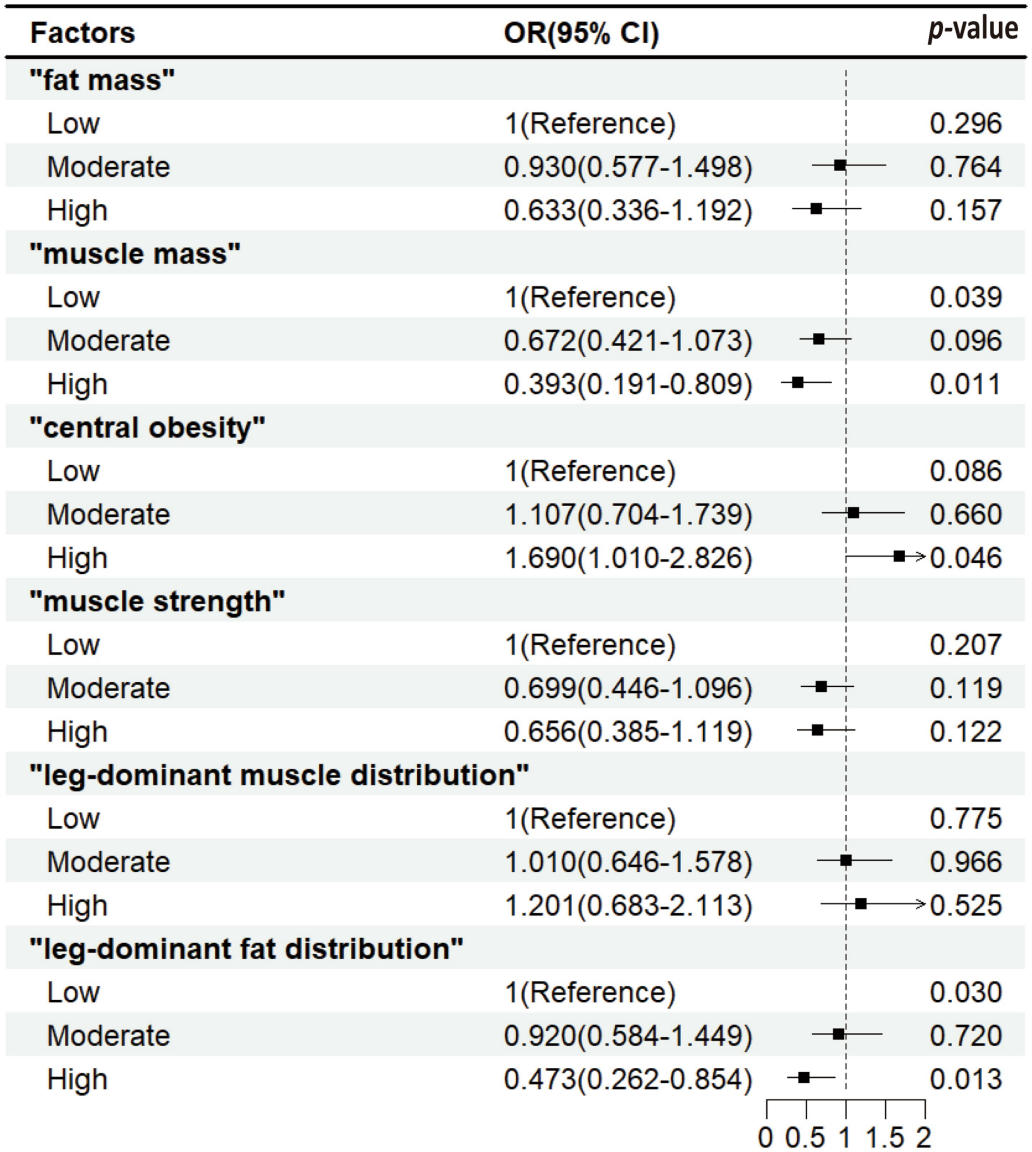
Associations between body composition and cognitive decline. Odds ratio (OR) and 95% confidence interval (CI) were calculated by the logistic regression model, with adjustments for age, gender, education, smoking and alcohol use, cardiovascular disease, rheumatic arthritis, and depressive symptoms.

Figure 1 shows that among the six factors, “central obesity,” “muscle mass,” and “leg-dominant fat distribution” were associated with cognitive decline. Compared with low exposure level, high exposure levels of “central obesity” (OR for high level 1.69 [1.01–2.826]) were associated with a higher rate of cognitive decline, while higher exposure levels of “muscle mass” (OR for high level 0.393 [0.191–0.809]) and “leg-dominant fat distribution” (OR for high level 0.473 [0.262–0.854]) were associated with a lower rate of cognitive decline.

### Network analysis

**Figure 2:**
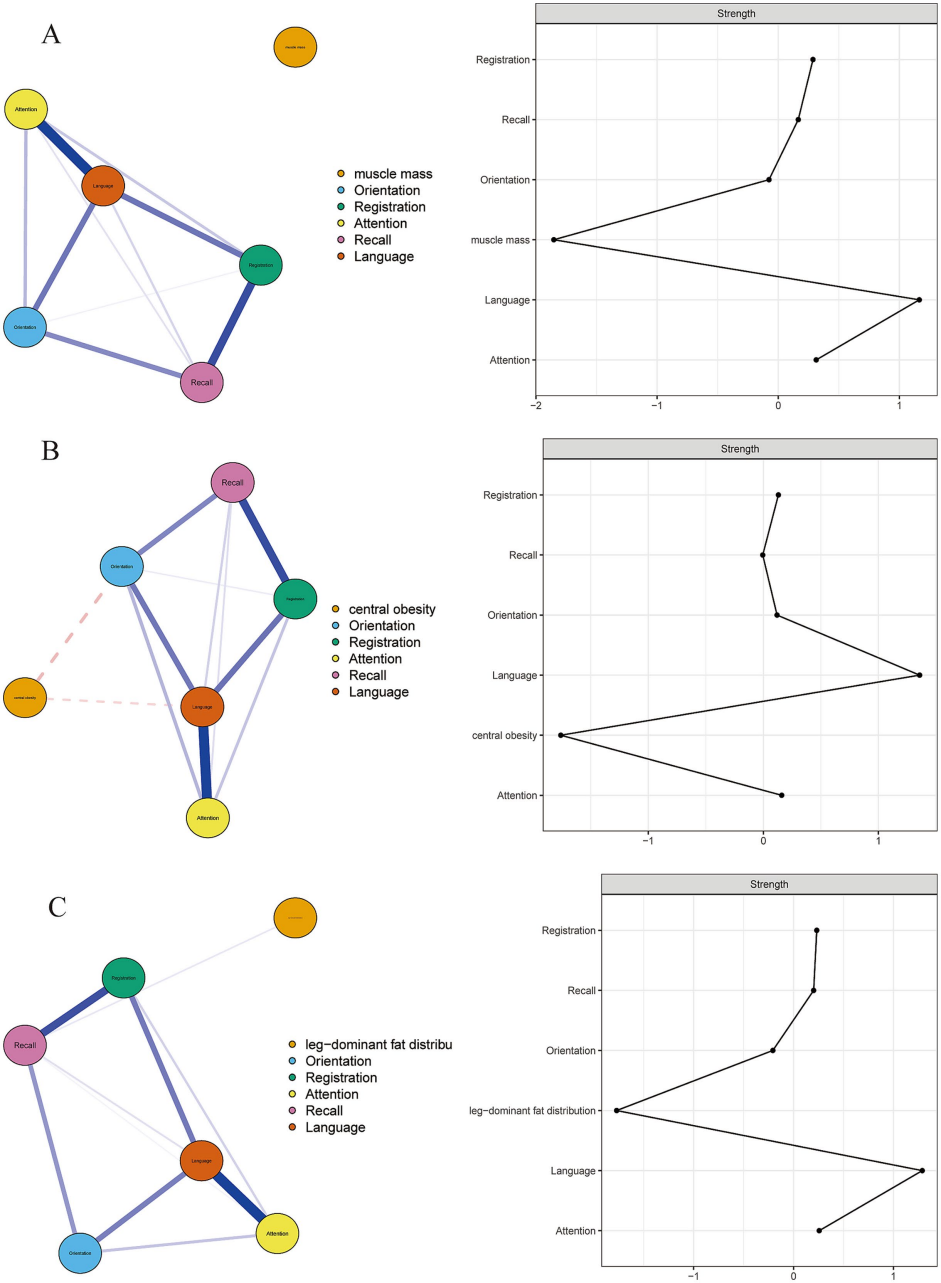
Network analysis of body composition and cognition domains in rural adults. The blue edges represent a positive correlation, and the red edges represent a negative correlation. The thickness of the edge corresponds to the size of the correlation, with thicker “edges” indicating a stronger correlation.

Figure 2A shows the network structure and centrality of “muscle mass” and cognition domains in rural adults. The “muscle mass” shows no significant correlation with any cognitive domains (edge weight = 0). Language (*z* = 1.16), attention (*z* = 0.31), and registration (*z* = 0.31) were the most central domains. Figure 2B shows the network structure and centrality of “central obesity” and cognition domains. The associations between “central obesity” and orientation or language were negative. Language (*z* = 1.35), attention (*z* = 0.15) and registration (*z* = 0.13) were the most central domains. Figure 2C shows the network structure and centrality of “leg-dominant fat distribution” and cognition domains. The associations between “leg-dominant fat distribution” and recall were positive. Language (*z* = 1.29), attention (*z* = 0.27) and registration (*z* = 0.23) were the most central domains. In terms of the stability and accuracy of primary network analysis, Supplementary Figure S1 shows that the CS-coefficient of strength ≥0.5, indicating the centrality values of networks were stable and accurate. In network analyses shown in Supplementary Figure S2, after controlling for covariates that might influence cognitive function, the most central cognitive domains remained similar to those found in the primary network analysis.

### Subgroup analysis

**Figure 3:**
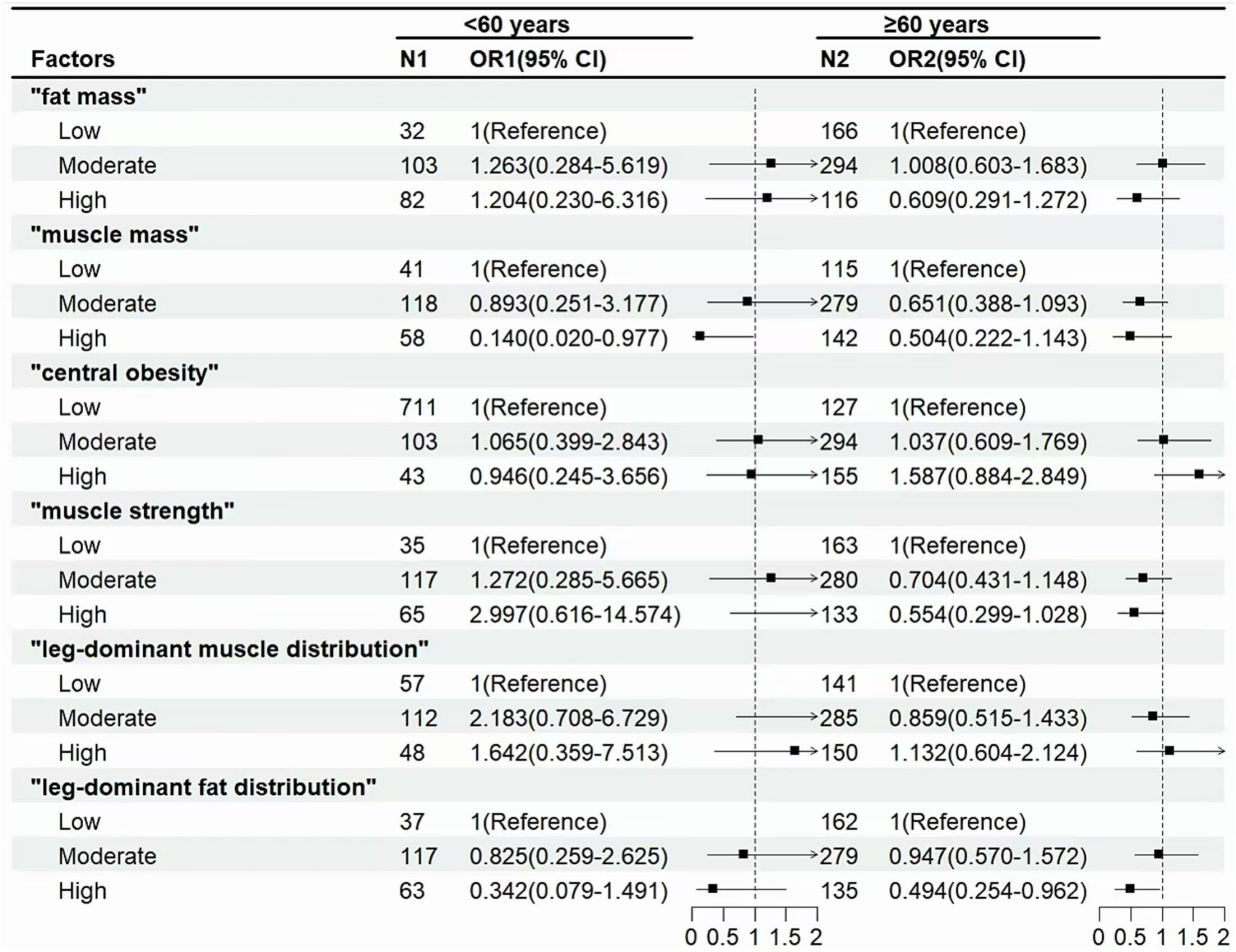
Associations between body composition and cognitive decline in non-elderly (<60) or elderly (≥60). Odds ratio (OR) and 95% confidence interval (CI) were calculated by the logistic regression model, with adjustments for age, gender, education, smoking and alcohol use, cardiovascular disease, rheumatic arthritis, and depressive symptoms.

Figure 3 shows that in the non-elderly population (<60 years), “muscle mass” (OR for high level: 0.14 [0.02–0.977]) was associated with a lower incidence of cognitive decline. In the elderly population (≥60 years), “leg-dominant fat distribution” (OR for high level: 0.494 [0.254–0.962]) was associated with a lower incidence of cognitive decline.

**Figure 4:**
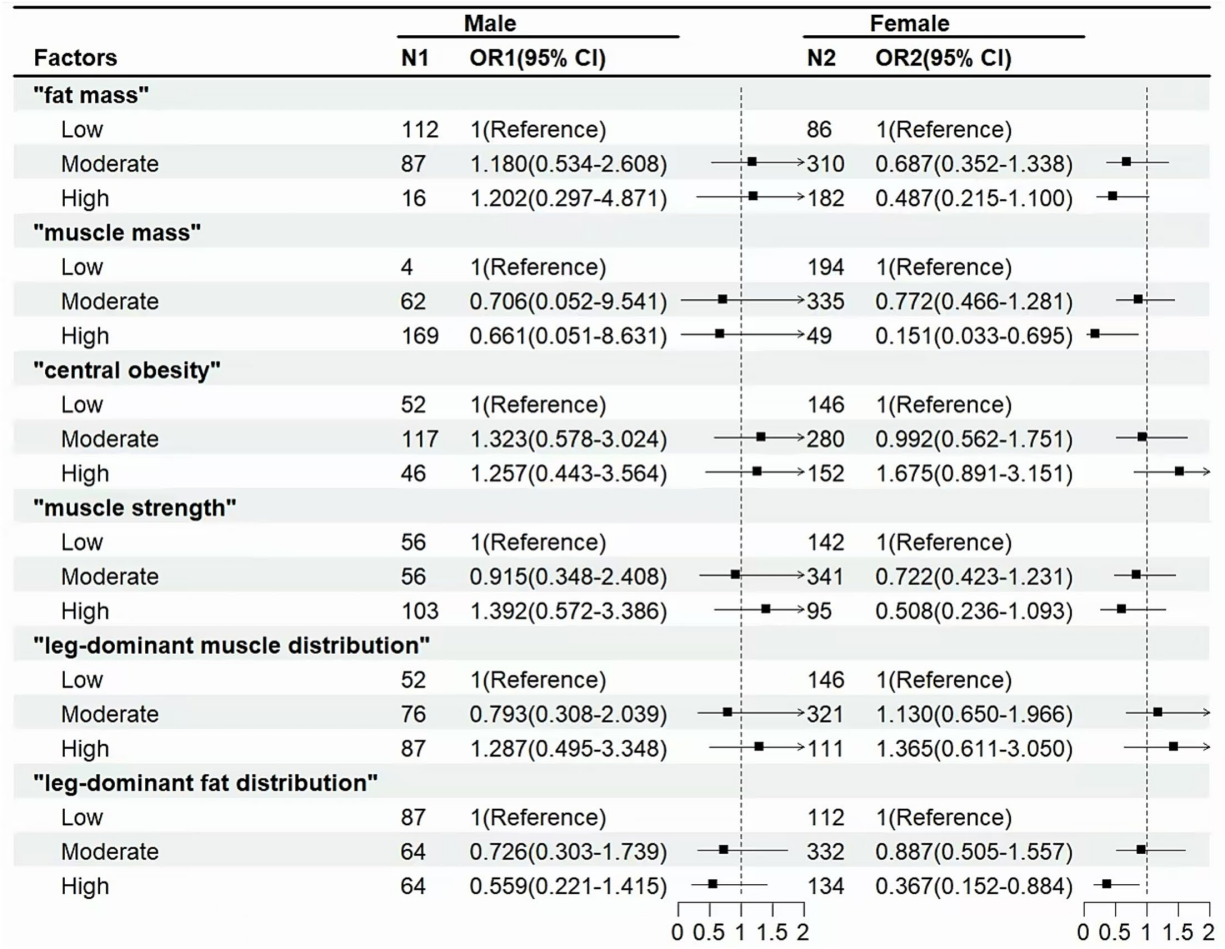
Associations between body composition and cognitive decline in male or female. Odds ratio (OR) and 95% confidence interval (CI) were calculated by the Logistic Regression Model, with adjustments for age, gender, education, smoking and alcohol use, cardiovascular disease, rheumatic arthritis, and depressive symptoms.

Figure 4 shows that in the female population, “muscle mass” (OR for high level: 0.151 [0.033–0.695]) and “leg-dominant fat distribution” (OR for high level: 0.367 [0.152–0.884]) were associated with a lower incidence of cognitive decline.

### Sensitivity analysis

Supplementary Table S3 shows similar results to those above, also supporting the findings of the quartile method. Moreover, compared with the dichotomous method and the tertile method, the quartile method had a better model fitting effect.

## Discussion

This cross-sectional study of 793 participants was conducted to screen for cognitive decline and to explore the relationship between body composition and cognitive function, considering interactions among body composition factors. Network analysis was used to analyze the key variables in this relationship for the first time.

Among the six body composition factors identified by exploratory factor analysis, “central obesity” was associated with an increased risk of cognitive decline, whereas “muscle mass” and “leg-dominant fat distribution” were associated with reduced risk. The findings are broadly consistent with previous research (Mina et al., 2023; Uchida et al., 2024). Previous studies have found that “muscle strength” plays a more critical role in the onset and progression of cognitive impairment than “muscle mass” (Beeri et al., 2021). Still, with the deepening of research, the importance of “muscle mass” in cognitive impairment has been gradually emphasized (Huang et al., 2025). The “leg-dominant fat distribution” can reduce the risk of cognitive decline. When body fat is preferentially concentrated in the legs, it means that less fat is concentrated in the abdomen and vital organs (e.g., liver, gallbladder, and pancreas). It is also observed that, in this factor, muscle mass is preferentially distributed in the limbs, which may be associated with regular high physical activity in rural populations. The above findings remind us to pay attention to the distribution of fat in the body. Targeted adjustment of fat distribution, along with regular exercise to increase muscle mass, may be an effective means to reduce the risk of cognitive impairment.

The mechanism of the relationship between body composition and cognitive decline remains unclear. However, the existing mechanisms primarily focus on critical pathways, including inflammatory response, metabolic disorders, and adipokine regulation. Ectopic fat secretes pro-inflammatory cytokines (TNF-*α*, IL-6, CRP), causing low-grade inflammation throughout the body. These inflammatory factors can activate microglia across the blood–brain barrier, leading to pro-inflammatory changes in the central nerves, such as the hypothalamus, impairing synaptic plasticity, and accelerating neuronal degeneration (Teixeira et al., 2023). Increased abdominal fat leads to the release of free fatty acids, which interfere with insulin signaling and trigger systemic insulin resistance (Shungin et al., 2015). Loss of muscle mass may induce insulin resistance through lipid infiltration, elevated branched-chain amino acid levels, and a decrease in the skeletal muscle type I ratio (Liu and Zhu, 2023). Additionally, some studies have found that insulin resistance is strongly associated with cognitive impairment, as assessed by cognitive function and brain imaging (Kim and Arvanitakis, 2023). In addition, the regulatory role of adipokines affects brain cognitive function to some extent. Adiponectin reduces inflammatory markers and enhances insulin sensitivity (Rizzo et al., 2020), whereas leptin resistance can induce cognitive impairment by activating inflammatory signals and causing endoplasmic reticulum stress in the hypothalamus (Flores-Cordero et al., 2022). Higher leg fat mass is associated with higher circulating adiponectin levels (Turer et al., 2011), so the “leg-dominant fat distribution” may prevent cognitive impairment by increasing adiponectin levels, reducing inflammatory markers, and improving insulin sensitivity.

“Muscle mass,” “central obesity,” and “leg-dominant fat distribution” were used to construct network models related to cognitive function. In these three models, the most central domains are all language, attention, and registration. Prior to our study, a study examined the relation between body composition and specific cognitive domains in women (Bove et al., 2013), consistent with our results, finding that language and registration are the core domains in the body composition and cognition relationship. A possible explanation is that language function involves a wide range of neural networks, which include the adjacent prefrontal, temporal, and parietal regions, as well as the bilateral caudate nucleus, the left nucleus pallidum, and thalamus primordium (Tomasi and Volkow, 2012). Systemic low-grade inflammation, caused by central obesity and loss of muscle mass (Teixeira et al., 2023), leads to inflammation in the Central Nervous System, disrupting the normal function of the neural network (Teipel et al., 2024). Language function involves the complex collaboration of multiple brain regions, and neuroinflammation can have wide-ranging effects on these regions and their connections, thereby reducing the efficiency and accuracy of language processing. Dopamine (DA) is a key neurotransmitter in the brain, and significant changes in attention occur when the midbrain cortical–limbic dopamine system is altered (Nieoullon, 2002). Obesity may impair dopaminergic neural pathways, which in turn affects attention and cognitive functioning in individuals (Epskamp et al., 2012). In addition, there is a bidirectional interaction between body composition and attention: a lack of attention leads to reduced self-regulatory control, which, in the long term, can result in abnormalities in body composition (Mann and Ward, 2007). Changes in body composition may induce insulin resistance (Snijder et al., 2004; Alser and Elrayess, 2022). Neuroimaging meta-analyses confirm that insulin resistance leads to enlarged perivascular spaces and increased synaptic connectivity deficits (Kim and Arvanitakis, 2023), with registration exhibiting high sensitivity to such neurostructural and functional alterations (Willette et al., 2015). Concurrently, as the most prevalent cognitive impairment in the elderly, Alzheimer’s disease is characterized in its early stages by memory impairment (Kamatham et al., 2024). This signifies that memory function serves both as an early indicator of cognitive decline and as a sensitive marker of metabolic abnormalities—such as insulin resistance—within the cognitive domain. In summary, alterations in body composition influence neural structure and function by inducing insulin resistance, thereby preferentially affecting memory function. This explains why registration constitutes the core domain within network models.

The subgroup analysis presented interesting results. In the non-elderly population, “muscle mass” had a notable protective effect, whereas in the elderly population, “leg-dominant fat distribution” did. As the largest organ involved in glucose metabolism, muscle tissue plays a crucial role in directly influencing brain energy supply and insulin sensitivity (Liu and Zhu, 2023). As muscle loss is an inevitable consequence of aging, the accumulation of fat may serve as a significant pathway through which body composition influences cognitive function in the elderly (Barazzoni et al., 2018). The concentration of fat in the legs plays a crucial role in mitigating ectopic fat deposition and reducing systemic inflammatory load, thereby providing protection for cognitive health (Mooldijk et al., 2023). This finding suggests that cognitive protection strategies should be dynamically adjusted with age, “muscle gain” in late middle age, “fat adjustment” in old age, and precise intervention in body composition at each stage. Besides, gender-stratified analysis revealed that the impact of body composition on cognition function is more pronounced in women, which may be related to women’s unique estrogen fluctuations (Russell et al., 2019; Boyle et al., 2021; Mosconi et al., 2024).

There are limitations to this study. First, this study uses a cross-sectional design, which can only capture associations and cannot determine causal relationships between body composition and cognitive impairment. Second, the sample is from rural areas. Although the focus is on characteristic populations, the representativeness is limited, and differences in urban and rural life and medical resources may affect body composition, so the sample should be expanded to include urban and multi-regional areas to extend the conclusions.

## Conclusion

In conclusion, this study explored the association between body composition and cognitive decline in rural adults through cross-sectional studies, and for the first time, used network analysis to reveal the key variables in this relationship. The results suggest that “central obesity” increases the risk of cognitive decline, whereas “muscle mass” and “leg-dominant fat distribution” exert protective effects. It provides a reference for the early prevention and control of cognitive decline among the elderly in rural areas. Additionally, interventions targeting language and registration domains may be beneficial in addressing cognitive decline resulting from changes in body composition.

## Data Availability

The raw data supporting the conclusions of this article will be made available by the authors, without undue reservation.
