# Supplementary material for: Body composition and cognitive function in Chinese rural adults: an exploratory factor analysis and network analysis
Source: Front Aging Neurosci. 2025 Dec 18;17:1722050. doi: 10.3389/fnagi.2025.1722050 (PMC12756470; doi:10.3389/fnagi.2025.1722050)
Supplement: Supplementary file 1 [file Data_Sheet_1.docx]

**Supplemental materials**

**1.Supplemental methods**

R script

**2. Supplemental figures and tables**

Table S1. Detailed information about 27 measurements of body composition 3

Table S2. Body composition index and factor loading matrix after rotation 4

Figure S1. Bootstrapped stability of primary network analysis 5

Figure S2. Network sensitivity analysis including body composition, cognition domains and demographic factors in rural adults 6

Table S3. Sensitivity Analyses for the relationship of body composition patterns with cognitive decline 7

**R script**

Construction and Visualisation of Variable Association Networks Based on the EBICglasso Algorithm

Function: Construct variable association networks using the Lasso sparsification method and visualise them according to predefined groupings

Packages and versions: qgraph (1.9.8), bootnet (1.6)

Grouping:

group <- list( "body composition" = 1, "Orientation" = 2, "Registration" = 3, "Attention" = 4, "Recall" = 5, "Language" = 6, "covariates" = 7:12 )

Network model estimation:

network2 <- estimateNetwork(data_select, default = "EBICglasso", tuning = 0.5,corMethod="cor", corArgs=list(method="spearman", use="pairwise.complete.obs"))

Network visualisation:

plot(network2, layout = "spring", groups = group, label.cex = 0.7, label.color = 'black', negDashed = TRUE, legend = TRUE,legend.cex = 0.4)

Network Model Summary (Number of Nodes, Number of Edges, Sparsity):

summary(network2)

Assessing Stability of Node Strength Using Bootstrap Method Function: Tests stability of network node strength via bootstrap resampling, calculates Correlation Stability coefficient (CS-coefficient)

Package: bootnet(1.6)

Centrality Stability Test:

set.seed(123)

Centrality_Stability2 <- bootnet(network2, boots=1000, nCores=8, type = "case",

statistics = c("Strength"))

Visualisation of stability results:

plot(Centrality_Stability2,statistics = c("Strength"))

Calculation of CS-coefficient:

corStability(Centrality_Stability2)

| **Measurement** | **Method/Meaning** |
| --- | --- |
| Waist circumference (WC, cm) | Use a flexible measuring tape to measure horizontally around the waist in a circle with the navel as the center. |
| Hip circumference (HC, cm) | Use a flexible measuring tape to measure horizontally around the buttocks in a circle, with the fullest part of the buttocks as the measurement point. |
| Waist-to-hip ratio (WHR, cm/cm) | WC (cm)/HC (cm) |
| Waist-to-height ratio (WHtR, cm/cm) | WC (cm)/height (cm) |
| Waist-to-weight index (WWI, cm/kg) | WC (cm)/ weight (kg)^0.5 |
| Body shape index (BSI, cm/kg) | WC (cm)/(BMI^2/3) *(height (m)^0.5) |
| Cone index(CI) | WC (cm)/10.9*((weight(kg)/(height (m))^0.5) |
| Grip strength (GS, kg) | maximum grip strength of right and left hands |
| Grip/BMI (kg/kg) | maximum grip strength/BMI |
| Grip/Arm Muscle Mass (Grip/AMM, kg/kg) | maximum grip strength/Arm Muscle Mass |
| Arm Fat Mass (AFM, kg) | Right Arm Fat Mass + Left Arm Fat Mass |
| Leg Fat Mass (LFM, kg) | Right Leg Fat Mass + Left Leg Fat Mass |
| Trunk Fat Mass (TFM, kg) | - |
| Total Body Fat Mass (TBFM, kg) | Arm Fat Mass + Leg Fat Mass +Trunk Fat Mass |
| Arm Muscle Mass (AFM, kg) | Right Arm Muscle Mass + Left Arm Muscle Mass |
| Leg Muscle Mass (LMM,kg) | Right Leg Muscle Mass + Left Leg Muscle Mass |
| Trunk Muscle Mass (TMM, kg) | - |
| Total Body Muscle Mass (TBMM, kg) | Arm Muscle Mass + Leg Muscle Mass + trunk Muscle Mass |
| Arm Fat Mass/Arm Muscle Mass (AFM/AMM, kg/kg) | Arm Fat Mass/Arm Muscle Mass |
| Leg Fat Mass/Leg Muscle Mass (LFM/LMM, kg/kg) | Leg Fat Mass/Leg Muscle Mass |
| Trunk Fat Mass/Trunk Muscle Mass (TFM/TMM, kg/kg) | Trunk Fat Mass/Trunk Muscle Mass |
| Arm Fat Mass/Total Body Fat Mass (AFM/TBFM, kg/kg) | Arm Fat Mass/Total Body Fat Mass |
| Leg Fat Mass/Total Body Fat Mass (LFM/TBFM, kg/kg) | Leg Fat Mass/Total Body Fat Mass |
| Trunk Fat Mass/Total Body Fat Mass (TFM/TBFM kg/kg) | Trunk Fat Mass/Total Body Fat Mass |
| Arm Muscle Mass/Total Body Muscle Mass (AMM/TBMM, kg/kg) | Arm Muscle Mass/Total Body Muscle Mass |
| Leg Muscle Mass/Total Body Muscle Mass (LMM/TBMM, kg/kg) | Leg Muscle Mass/Total Body Muscle Mass |
| Trunk Muscle Mass/Total Body Muscle Mass (TMM/TBMM, kg/kg) | Trunk Muscle Mass/Total Body Muscle Mass |

**Table S1. Detailed information about 27 measurements of body composition**

**Table S2. Body composition index and factor loading matrix after rotation**

| Body composition | Factor1 | Factor2 | Factor3 | Factor4 | Factor5 | Factor6 |
| --- | --- | --- | --- | --- | --- | --- |
| TBFM (kg) | 0.966 |  |  |  |  |  |
| AFM (kg) | 0.963 |  |  |  |  |  |
| LFM (kg) | 0.959 |  |  |  |  |  |
| TFM (kg) | 0.944 |  |  |  |  |  |
| AMM/TBMM (kg/kg) | -0.875 |  |  |  |  |  |
| AFM/AMM (kg/kg) | 0.864 |  |  |  |  |  |
| TFM/TMM (kg/kg) | 0.846 |  |  |  |  |  |
| LFM/LMM (kg/kg) | 0.804 |  |  |  |  |  |
| HC (cm) | 0.677 |  |  |  |  |  |
| WHtR (cm) | 0.653 |  |  |  |  |  |
| WC (cm) | 0.582 |  |  |  |  |  |
| AMM (kg) |  | 0.92 |  |  |  |  |
| LMM (kg) |  | 0.978 |  |  |  |  |
| TMM (kg) |  | 0.882 |  |  |  |  |
| TBMM (kg) |  | 0.982 |  |  |  |  |
| AFM/TBFM (kg/kg) |  | -0.762 |  |  |  |  |
| WHR (cm/cm) |  |  | 0.78 |  |  |  |
| WWI (cm/kg) |  |  | 0.816 |  |  |  |
| BSI (cm/kg) |  |  | 0.907 |  |  |  |
| CI |  |  | 0.935 |  |  |  |
| GS (kg) |  |  |  | 0.903 |  |  |
| GS/AMM (kg/kg) |  |  |  | 0.893 |  |  |
| GS/BMI (kg/kg) |  |  |  | 0.874 |  |  |
| LMM/TBMM (kg/kg) |  |  |  |  | 0.768 |  |
| TFM/TBFM (kg/kg) |  |  |  |  | 0.691 |  |
| TMM/TBMM (kg/kg) |  |  |  |  |  | -0.767 |
| MFM/TBFM (kg/kg) |  |  |  |  |  | 0.578 |

Abbreviation: TBFM, Total body fat mass; AFM, Arm fat mass; LFM, Leg fat mass; TFM, Trunk fat mass; AMM/TBMM, Arm muscle mass/total body muscle mass; AFM/AMM, Arm fat mass/arm muscle mass; LFM/LMM, Leg fat mass/leg muscle mass; TFM/TMM, Trunk fat mass/trunk muscle mass; HC, Hip circumference; WHtR, Waist-to-height ratio; WC, Waist circumference; AMM, Arm muscle mass; LMM, Leg muscle mass; TMM, Trunk muscle mass; TBMM, Total body muscle mass; AFM/TBFM, Arm fat mass/total body fat mass; WHR, Waist-to-hip ratio; WWI, Weight-adjusted waist circumference index; BSI, Body shape index; CI, Cone index; GS, Grip strength; GS/AMM, Grip strength/arm muscle mass; GS/BMI, Grip strength/BMI; LMM/TBMM, Leg muscle mass/total body muscle mass; TFM/TBFM, Trunk fat mass/total body fat mass; TMM/TBMM, Trunk muscle mass/total body muscle mass; LFM/TBFM, Leg fat mass/total body fat mass;

**Figure S1. Bootstrapped stability of primary network analysis****
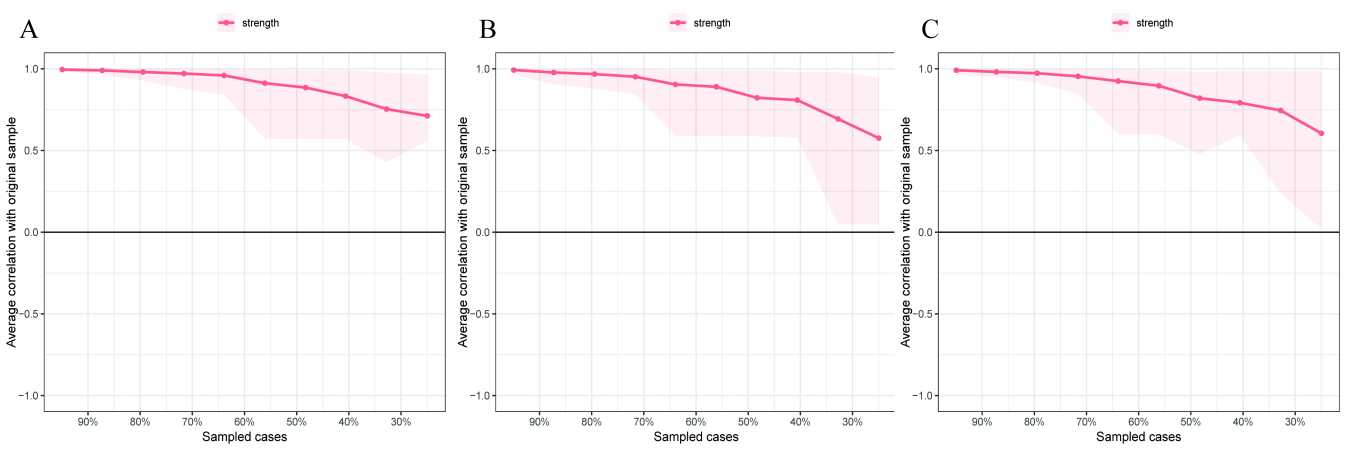
**

Figure S1.A shows the CS-coefficient of “muscle mass” and cognition domains in rural adults. Figure S1.B shows the CS-coefficient of “central obesity” and cognition domains. Figure S1.C shows the CS-coefficient of “leg-dominant fat distribution” and cognition domains.

**Figure S2. Network sensitivity analysis including body composition, cognition domains and demographic factors in rural adults**


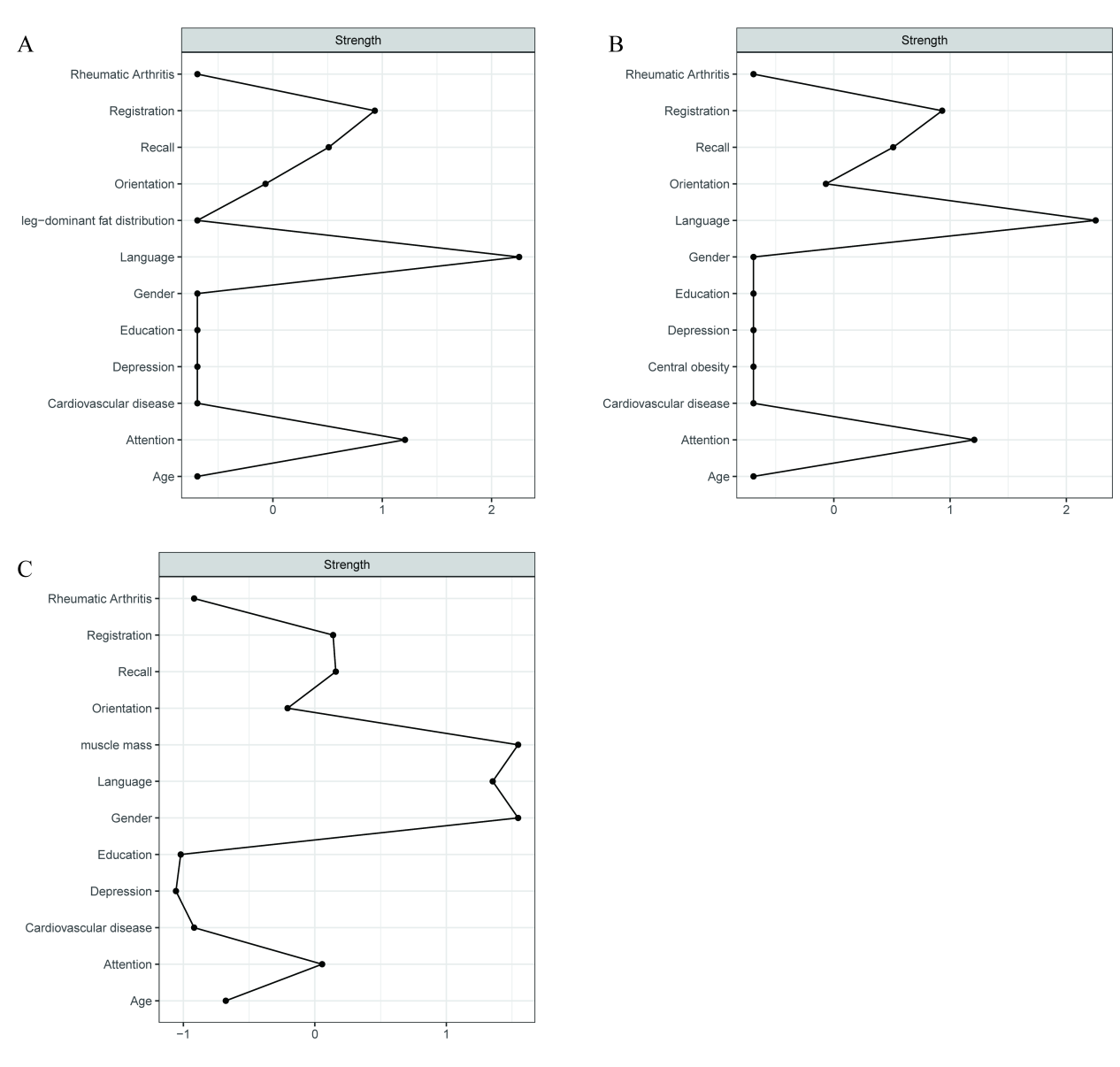


Figure S2.A shows the code centrality of “muscle mass” and cognition domains in rural adults. Figure S2.B shows the code centrality of “central obesity” and cognition domains. Figure S2.C shows the code centrality of “leg-dominant fat distribution” and cognition domains.

**Table S3. Sensitivity Analyses for the relationship of body composition patterns with cognitive decline**

| Factors | Median method | Tertile method | Quartile Method |
| --- | --- | --- | --- |
| "fat mass" |  |  |  |
| Low | 1(Reference) | 1(Reference) | 1(Reference) |
| Moderate | NA | 0.862(0.544-1.365) | 0.93(0.577-1.498) |
| High | 0.903(0.608-1.342) | 0.878(0.513-1.503) | 0.633(0.336-1.192) |
| "muscle mass" |  |  |  |
| Low | 1(Reference) | 1(Reference) | 1(Reference) |
| Moderate | NA | 0.753(0.471-1.204) | 0.672(0.421-1.073) |
| High | 0.796(0.516-1.23) | 0.601(0.327-1.106) | 0.393(0.191-0.809) |
| "central obesity" |  |  |  |
| Low | 1(Reference) | 1(Reference) | 1(Reference) |
| Moderate | NA | 1.064(0.682-1.66) | 1.107(0.704-1.739) |
| High | 1.282(0.895-1.839) | 1.32(0.84-2.075) | 1.69(1.01-2.826) |
| "muscle strength" |  |  |  |
| Low | 1(Reference) | 1(Reference) | 1(Reference) |
| Moderate | NA | 0.833(0.534-1.3) | 0.699(0.446-1.096) |
| High | 0.781(0.541-1.128) | 0.764(0.483-1.209) | 0.656(0.385-1.119) |
| "leg-dominant muscle distribution" | |  |  |
| Low | 1(Reference) | 1(Reference) | 1(Reference) |
| Moderate | NA | 0.869(0.555-1.361) | 1.01(0.646-1.578） |
| High | 1.062(0.732-1.541) | 1.098(0.682-1.765) | 1.201(0.683-2.113) |
| "leg-dominant fat distribution" | |  |  |
| Low | 1(Reference) | 1(Reference) | 1(Reference) |
| Moderate | NA | 0.855(0.548-1.334) | 0.92(0.584-1.449) |
| High | 0.709(0.488-1.03) | 0.529(0.322-0.869) | 0.473(0.262-0.854) |
| Model fit statistics |  |  |  |
| AUC | 0.674 | 0.683 | 0.7 |
| AIC | 799.379 | 793.99 | 783.224 |
| BIC | 892.379 | 887.49 | 876.644 |

NA, not applicable (the median method does not have a moderate exposure category).
